# Sanitation, Disease Externalities and Anaemia: Evidence From Nepal

**DOI:** 10.1111/ecoj.12491

**Published:** 2017-08-07

**Authors:** Diane Coffey, Michael Geruso, Dean Spears

**Affiliations:** ^1^ University of Texas at Austin, Indian Statistical Institute; ^2^ University of Texas at Austin and NBER

## Abstract

Anaemia impairs physical and cognitive development in children and reduces human capital accumulation. The prior economics literature has focused on the role of inadequate nutrition in causing anaemia. This article is the first to show that sanitation, a public good, significantly contributes to preventing anaemia. We identify effects by exploiting rapid and differential improvement in sanitation across regions of Nepal between 2006 and 2011. Within regions over time, cohorts of children exposed to better community sanitation developed higher haemoglobin levels. Our results highlight a previously undocumented externality of open defaecation, which is today practiced by over a billion people worldwide.

Anaemia is a widespread problem with serious health and economic consequences. Defined by low counts of red blood cells or low levels of haemoglobin in the bloodstream, anaemia implies a reduced capacity for the blood to carry oxygen. In adults, it reduces productivity (Thomas *et al*., 2004) and is associated with higher maternal mortality (Rush, 2000). In children, it impairs physical and cognitive development directly (Grantham‐McGregor and Ani, 2001; Ozier, 2015) and affects human capital accumulation via impacts on behaviour such as school attendance (Bobonis *et al*., 2006). Globally, more than 40% of children have haemoglobin levels below the threshold for anaemia.^1^ The problem is particularly severe in the developing world, as anaemia is closely associated with inadequate nutrition.

Because of its damaging effects on human capital formation and productivity, anaemia has attracted significant research and policy attention. Economic research in the area of preventing or reducing anaemia has generally focused on:

- poor nutrition, and in particular iron deficiency (Bhattacharya *et al*., 2004; Thomas *et al*., 2004); and
- malaria (Sachs and Malaney, 2002), which is a parasitic infection that attacks the red blood cells.

Nonetheless, there are reasons to believe that poor nutrition and malarial disease are not the only important causes of anaemia. For one, international variation in anaemia rates is not well explained by international variation in income (Alderman and Linnemayr, 2009). To the extent that income is a reasonable proxy for basic nutrition, this poses somewhat of a puzzle. Second, although it is well known that in sub‐Saharan Africa malaria is a major cause of anaemia, anaemia rates are the highest in South Asia, where malaria is far less prevalent.

In this article, we propose a third broad cause of anaemia that operates in addition to nutritional intake and malaria, and which has important (and different) implications for policy. We propose that poor local sanitation causes lower haemoglobin levels and higher rates of anaemia in children. Following recent literature (Guiteras *et al*., 2015), we operationalise poor sanitation by measuring open defaecation, which is defaecation outside on the open ground, without the use of a toilet or latrine. Whereas nutritional intake is a behaviour with purely private benefits, poor sanitation primarily constitutes an external harm: it spreads faecal pathogens across individuals, since these are transmitted by contact with the faecal matter that is left in the open. As we discuss below, there is significant epidemiological evidence suggesting that sanitation could play an important role in determining anaemia. Among other channels, the intestinal parasites and other infections spread by open defaecation can affect the intestinal wall in ways that lead to decreased absorption of nutrients, including iron, vitamin B12 and folic acid (Rosenberg and Bowman, 1982; Nath, 2005), which are critical for the production of haemoglobin. If sanitation were indeed an important determinant of haemoglobin levels, its potential role in the worldwide, aggregate patterns of anaemia would be staggering: more than a billion people (about 14% of the world's population) defaecate in the open today.

Ours is the first article to empirically link open defaecation to anaemia. Nevertheless, the possibility of such a link is suggested by a chain of evidence in a small body of prior work. With respect to the connection between open defaecation and intestinal parasites, a randomised control trial in Indonesia that included toilet construction and behaviour change interventions to discourage open defaecation found evidence that reduced rates of open defaecation were associated with reduced intestinal parasite infections (Cameron *et al*., 2013). With respect to the connection between intestinal parasite infections and anaemia, a randomised control trial among Kenyan children found that a single dose of intestinal parasite (deworming) medicine was as effective in improving haemoglobin levels as a daily supplement of 13 micronutrients including iron taken for eight months (Friis *et al*., 2003).^2^ Miguel and Kremer (2004) also provide experimental evidence that deworming reduces anaemia in the Kenyan context. The clearest evidence to date suggesting that poor local sanitation may cause anaemia is presented in Bleakley (2007), which studied the effects of a hookworm eradication campaign in the US South at the turn of the twentieth century. The eradication efforts included the construction and promotion of sanitary latrines. That paper found large effects on school attendance and later‐life earnings for children exposed to the eradication campaign. The hypothesised mechanism was reduced anaemia, but the historical data could not be used to provide direct evidence of an effect of the sanitary environment on blood haemoglobin levels.^3^

We examine the impact of sanitation on children's haemoglobin levels in the context of Nepal. Nepal is an ideal empirical setting for several reasons. First, Nepal has very little malaria, which in other developing country contexts could be an important confounder when examining anaemia. Second, the Nepal DHS surveys – unlike, for example, the Indian DHS or other Indian data sets – have collected blood haemoglobin measurements over multiple survey waves and report geographic identifiers at a relatively disaggregated level, allowing us to create a geographic panel with anaemia measures.^4^ Finally, Nepal has had relatively high rates of open defaecation historically but also rapid improvement in sanitation in the recent past. In 2006, about 50% of Nepali households defaecated in the open – that is, they reported, using a bush, field or no facility. By this measure, Nepali households faced among the worst sanitation environments in the world. The rates of open defaecation in Nepal were worse, for example, than in most countries in sub‐Saharan Africa at the time.

However, following the introduction of national government initiatives aimed at reducing open defaecation, there was a rapid improvement in latrine and toilet use. By 2011, the fraction of households defaecating in the open had declined to a national mean of 35%, with significant variation in improvements across regions. The effective ‘exposure’ of a locality to government‐led sanitation efforts in the mid 2000s was heavily constrained by the then‐current level of sanitation in each locality. Places with historically worse sanitation had a larger scope for improvement in level terms. For example, regions that were already open defaecation free by 2006 could experience no further improvements, while regions with the highest open defaecation rates (as high as 70%) experienced the largest level changes (in excess of 30 percentage points) over our short panel.

We exploit the geographically heterogeneous sanitation improvements from 2006 to 2011 to identify impacts of poor sanitation on haemoglobin in difference‐in‐differences regressions. We find that cohorts of children exposed to better community sanitation developed higher haemoglobin levels. Controlling for own defaecation practice, a 10 percentage point decrease in the fraction of neighbours who defaecate in the open is associated with a 0.13 g/dL increase in haemoglobin levels, or about 9% of a standard deviation. To put this effect size in context, interventions in the experimental nutrition literature, such as micronutrient supplementation (Friis *et al*., 2003), iron supplementation (Lind *et al*., 2003) and iron fortification in foods (Van Stuijvenberg *et al*., 1999), have effect sizes that range from 0.20 to 0.41 g/dL. The effect sizes we estimate are consistent with the experimental evidence on the efficacy of anti‐intestinal parasite interventions by Friis *et al*. (2003), in which a single dose of deworming medicine increased haemoglobin by 0.21 g/dL. As we describe in greater detail below, the spread of intestinal worms via contact with faecal matter comprises just one of the channels by which the effects we find are likely to operate.

The parallel trends assumption underlying our difference‐in‐differences analysis is that regional variation in sanitation improvements was not correlated with other changes within regions over time that could independently affect haemoglobin levels. The list of potential confounding variables is somewhat narrowed by the extensive prior economic, epidemiological and medical literature on the causes of anaemia. Our data allow us to directly test for parallel trends in variables that span this small set of plausible confounding factors. We show that diet, the consumption of iron supplements and the use of deworming treatments were not differentially trending in places with greater sanitation improvement. Further, we provide evidence that our results are not driven by a broader package of changes in the local physical infrastructure that were spuriously correlated with improvements in latrine availability.

The main contribution of our study is to advance the basic scientific understanding of the causes of anaemia. Anaemia has attracted significant attention as a human capital outcome in the US and the developing world (Bhattacharya *et al*., 2004; Miguel and Kremer, 2004; Thomas *et al*., 2004; Cohen and Dupas, 2010), though prior efforts by economists to identify the determinants of anaemia (Thomas *et al*., 2004; Bobonis *et al*., 2006) have often focused on inputs constituting private goods. In particular, past experimental interventions have randomised whether an individual child received an iron supplement, fortified food, or deworming medicine. Our study is unique in investigating a public goods cause of anaemia and thus complements this existing literature.^5^ More broadly, we view our results as contributing to an expanding understanding of what constitutes nutrition. Anaemia is typically labelled a ‘nutritional’ outcome but we show here that it is affected by a disease environment that impairs nutrient absorption, rather than affecting nutrient intake. In this way, our findings connect to the wider literature on the importance of nutrition during early childhood for human capital accumulation (Maluccio *et al*., 2009; Deaton, 2013) and are consistent with recent work by Duh and Spears (2017) on the importance of considering net nutrition, rather than merely calorie intake.^6^

Our finding that the local sanitation environment is a public good affecting haemoglobin suggests new policy avenues for addressing anaemia and raises new considerations for future research. One such policy implication is that reducing anaemia in children can in part be accomplished by changing the health behaviour of community members (i.e. neighbours) who are neither children nor parents. Improving sanitation raises its own set of difficulties exactly because sanitation is a public good and therefore subject to inadequate private investment (Guiteras *et al*., 2014), suggesting a welfare‐improving role for government. With respect to future research, our findings imply that any investigation of the role of open defaecation in determining anaemia requires variation that arises at the level of a neighbourhood or region (as it does in our empirical analysis), rather than at the person‐level. This suggests that the kind of cluster‐randomised trials that have recently been fielded to examine other aspects of local sanitation (Clasen *et al*., 2015; Guiteras *et al*., 2015) are also the right approach for future experimental work that builds on our findings regarding anaemia.

Finally, this article contributes to a growing literature concerned with the adverse health and human capital consequences of poor sanitation in the developing world. Open defaecation, in particular, has attracted significant policy attention and NGO investment in recent years for reasons unrelated to anaemia. Our findings on children's haemoglobin strengthen the rationale for such investments and may play a role in explaining some of the recent findings in the literature. For example, Spears and Lamba (2016) find that exposure to open defaecation negatively impacts child cognitive function, which is an outcome known to be affected by anaemia (Stoltzfus *et al*., 1997).

The remainder of the article is organised as follows. Section 1 discusses the known causes of anaemia and reviews the existing epidemiological evidence of a channel from poor sanitation to lower haemoglobin. Section 2 presents some new stylised facts from international comparisons that are intended to motivate our main analysis. Section 3 describes our data, identifying variation, and empirical strategy. Section 4 reports results, and Section 5 traces out the significance and policy relevance of our findings. Section 6 concludes.

## 1 Background on Anaemia and Sanitation

Haemoglobin is a protein which resides in red blood cells and which binds to iron in order to attract oxygen and carry it throughout the body. Iron deficiency anaemia is defined by haemoglobin below a threshold level. There are several known causes of low haemoglobin. These involve either too little production or too much destruction of haemoglobin.

Poor diets, particularly among young children, are an often‐cited cause of anaemia in developing countries (Yip and Ramakrishnan, 2002; Tolentino and Friedman, 2007). Although a major cause of low haemoglobin production is iron deficiency in the diet, low haemoglobin can also be caused by lack of vitamin B12 and folic acid, two nutrients necessary for the production of red blood cells. The late introduction of solid foods in infants and diets containing inadequate amounts of these essential nutrients are both important contributors to low haemoglobin in South Asia (Menon, 2012), the region of the developing world we study here.^7^

Malaria is another important cause of anaemia, particularly in sub‐Saharan Africa. The disease is transmitted by a mosquito bite, during which a parasitic protozoa carried by the mosquito enters the person's bloodstream. The malaria parasite attacks red blood cells, which are in turn attacked by the host's immune system. This destruction of red blood cells leads to anaemia. (The protozoan malaria parasite is significantly different in form, life cycle and symptomatic effects from the intestinal worms we discuss below, which are transmitted by contact with human excreta.)

How could poor sanitation affect anaemia? There are two plausible channels. The first is related to intestinal parasites and the second is a condition known as environmental enteropathy.^8^ Our study does not attempt to distinguish between these two epidemiological pathways, as both are consistent with an impact of poor sanitation and both are likely to be operating simultaneously.

Intestinal parasites are known to cause anaemia by causing blood loss in the stool, lack of appetite, increased motility of food through the intestine, and competition for nutrients.^9^ Intestinal parasites also cause damage to the intestinal wall that leads to decreased absorption of nutrients, including iron, vitamin B12 and folic acid (Rosenberg and Bowman, 1982). It has long been known that open defaecation spreads intestinal parasites; Cairncross (2003) cites research from the 1930s that describes how variation in community latrine use in the southern United States predicted parasite infections in children.^10^

The second pathway from open defaecation to haemoglobin levels is environmental enteropathy, known as tropical sprue in an older medical literature. It is a disease which alters the lining of the intestine and inhibits absorption of calories and nutrients. Although the link between open defaecation and enteropathy is less well understood than the link between open defaecation and intestinal worms, it is hypothesised that open defaecation exposes people to the kinds of bacteria that, when ingested in large quantities, lead to decreased absorption of micronutrients necessary for the production of haemoglobin (Walker, 2003; Nath, 2005; Humphrey, 2009).^11^ Medical researchers have hypothesised a link between enteropathy and anaemia as long ago as the 1920s (Baumgartner and Smith, 1927).

## 2 Stylised Facts from International Comparisons

**Figure 1:**
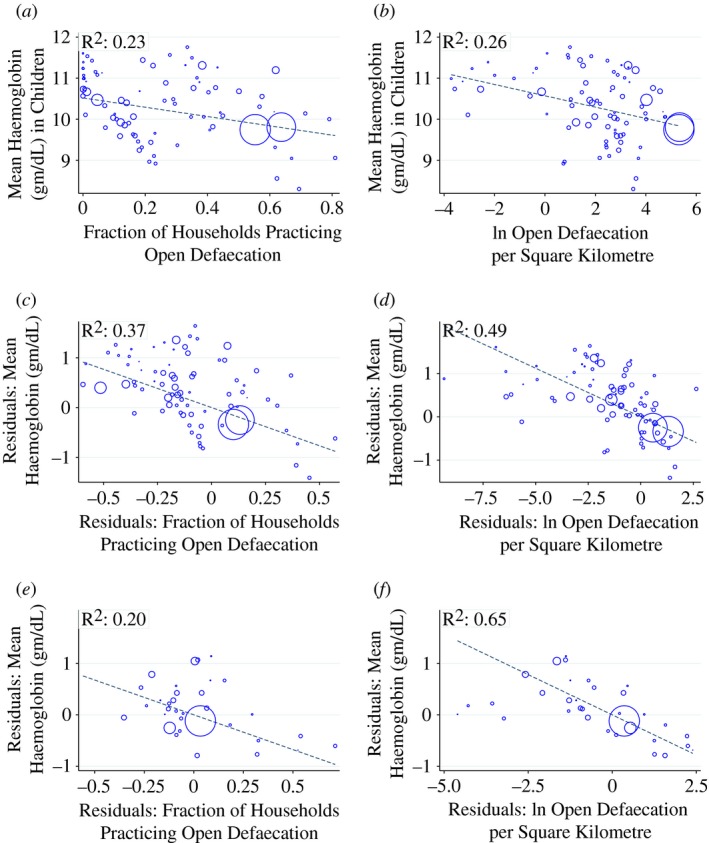
Motivating Facts: Cross‐country Relationships Between Haemoglobin and Open Defaecation. (a) and (b) Unconditional Correlation. (c) and (d) Controlling for Income and Malaria. (e) and (f) Controlling for Diet, Income, Malaria *Notes*. The Figure shows the cross‐country relationship between fraction of households practising open defaecation and haemoglobin levels in children aged 6–35 months. Each point in the scatterplots corresponds to a country × year mean of a Demographic and Health Survey. Marker sizes are proportional to country populations. Panels (*a*)–(*d*) contain 81 DHS surveys. Panels (*e*) and (*f*) contain the subset of 35 DHS surveys for which dietary information was available. See Table A1 for a list of the DHS surveys represented in the plots. Dashed lines in each plot correspond to regression coefficients from population‐weighted regressions. Colour figure can be viewed at http://wileyonlinelibrary.com

To motivate the our econometric analysis below, we begin in Figure 1 by documenting some cross‐country summary statistics relating sanitation to anaemia. To our knowledge, ours is the first study to document these patterns, even cross‐sectionally.

Data on anaemia and open defaecation used in the Figure comes from 81 Demographic and Health Surveys (DHS), covering 45 countries in sub‐Saharan Africa, Latin America, Europe, and Asia. The country × years that make up individual observations are listed in Table A1. The DHS are nationally representative surveys that collect information on health behaviour and outcomes of household members, including data on toilet use. In our data, if a respondent household reports using a ‘bush, field or no facility’, the household is coded as defaecating in the open. Following Spears (2013) we capture the sanitation environment to which a child is exposed by calculating the fraction of households that defaecate in the open. The greater the fraction of households that do not use a toilet or latrine, the greater the frequency with which a child comes into contact with germs or parasites transmitted by faeces.

Observations in Figure 1 are means from surveys (countries × years) and we include every survey for which data on children's haemoglobin was measured. The DHS measures haemoglobin using the HemoCue${}^{®}$ method, in which a surveyor introduces a drop of blood from the respondent's finger into a portable device that reports the respondent's haemoglobin level in the field.^12^

In panel (*a*) of Figure 1, we plot the unconditional relationship between the average haemoglobin level of children and the mean open defaecation rate in the country. We restrict attention to children aged 6–35 months, as this was the common age range for which haemoglobin data were recorded across the 81 DHS surveys represented in the Figure. The size of markers is proportional to country population and many countries appear twice in the scatterplot for different survey years. The Figure also plots a regression line corresponding to the population‐weighted OLS coefficient of open defaecation on haemoglobin. The plot shows a clear association in which more open defaecation (i.e. worse sanitation) predicts lower haemoglobin levels of children.

In panel (*b*), we modify the horizontal axis so that it measures the log of open defaecation per Square Kilometre, following recent literature showing that the risk of transmitting pathogens via open defaecation is increasing in population density (Spears, 2013; Hathi *et al*., 2014). Data on total land area and population, which are used to construct the measure of open defaecation per Square Kilometre, come from the Penn World Tables. The clear negative relationship continues to hold. While the slopes are not directly comparable between panels (*a*) and (*b*) since the horizontal axes are different, the overall decline in haemoglobin levels between observations with the best sanitation (leftmost points) and worst sanitation (rightmost points) are similar across the two panels.

A natural question in this context is whether the places with worse sanitation are merely worse in other ways that would independently predict anaemia. In particular, malaria incidence, which impacts anaemia, may be worse in countries where the sanitation environment is worse. And nutritional intake, which is the leading known cause of anaemia, may be worse where sanitation is worse, since both are correlated with income. In panels (*c*) and (*d*) of Figure 1, we control for malaria incidence using national malaria rates constructed by Korenromp (2005) and we control for GDP *per capita* using data from the Penn World Tables.^13^ In panels (*e*) and (*f*) of Figure 1, we additionally control for available diet information. The diet module is only available in a subset of the surveys (35 DHS country × years). For these surveys, respondents provide information about the types and amounts of foods consumed by their young children over the last 24 hours. The dietary controls include an indicator for the child consuming meat and eggs in the last 24 hours, an indicator for the child consuming fruits or vegetables in the last 24 hours and a dietary diversity measure, defined as the number of different kinds of foods consumed by the child in the last 24 hours.^14^ To display scatter plots that use the various control sets, we first separately regress haemoglobin and open defaecation on the indicated controls. We then plot the residuals from those two regressions against each other. The relationship between sanitation and anaemia is, in fact, stronger after the inclusion of controls for malaria and GDP *per capita*, as indicated by the R${}^{2}$ that is reported in the upper left of each panel. The addition of the dietary controls in panels (*e*) and (*f*) generate residual scatterplots that continue to show a clear relationship between OD and blood haemoglobin levels. Panel (*f*) is particularly striking, with observations tightly clustered around the regression line and the highest R${}^{2}$ of any panel.

Although Figure 1 is intended only to provide motivation for the econometric analysis below, the patterns it reveals are consistent with a previous finding in the literature that international variation in anaemia rates is not well explained by international variation in income (Alderman and Linnemayr, 2009). The patterns are also consistent with the fact that although malaria is less prevalent in South Asia than in sub‐Saharan Africa, rates of anaemia in South Asian countries often exceed those of sub‐Saharan African countries. In South Asia, open defaecation is more prevalent.

In summary, the cross‐country comparison reveals an interesting and previously undocumented pattern. Poor sanitation strongly predicts low haemoglobin, both unconditionally and controlling for income, measures of food intake, and malaria incidence. The remainder of the article investigates a causal relationship, using variation in open defaecation that is plausibly exogenous to haemoglobin levels.

## 3 Data and Empirical Framework

We investigate the hypothesised link between sanitation and anaemia using data from Nepal. Nepal ranks among the worst sanitation environments in the world. As recently as 2006, half of Nepali households disposed of excreta in the open, without the use of a toilet or latrine. But sanitation in Nepal has improved rapidly since that time, following sanitation initiatives launched by Nepal's central government in the mid‐2000s. The DHS data show a 13 percentage point decline in open defaecation at the national level between 2006 and 2011. Nepal's poor baseline sanitation, rapid improvement and very low rates of malaria, make it an ideal empirical setting for our study.

### 3.1 Data

The data used in our main analysis come from the 2006 and 2011 Demographic and Health Surveys (DHS) of Nepal.^15^ As described above, the DHS surveys are designed to be nationally representative. In addition to the variables used to construct Figure 1, mothers in these Nepal DHS rounds report on whether they took iron supplements during their most recent pregnancy and on whether each child in the sample has taken intestinal parasite medication in the last six months.

Typically, anaemia is defined by haemoglobin levels below some threshold value. The World Health Organization (WHO) sets the haemoglobin concentration threshold at 11 g/dL and 7 g/dL for anaemia and severe anaemia, respectively, though various researchers and medical bodies may set alternative cutoffs.^16^ In order to maximise power and avoid sensitivity to the choice of threshold, we use the entire continuous range of haemoglobin concentration in our main results, though we also report specifications in which the dependent variables are indicators for various anaemia thresholds.

From the measures of open defaecation, we generate variables capturing the mean open defaecation at the level of a neighbourhood or region, depending on the analysis. Neighbourhoods are reflected in the data by primary sampling units (PSUs), which are composed of approximately 100–200 co‐located households. In rural areas these PSUs may be whole villages. In urban areas, the PSUs are defined by blocks of households. Because the DHS does not re‐interview the same PSUs in different survey waves, in most specifications we aggregate location data to the smallest geographic unit for which it is possible to construct a panel in our data. The survey identifies 13 geographic areas in Nepal. We interact the urban indicator with indicators for each of 13 areas to create 25 ‘region’ indicators, which we exploit in our difference‐in‐differences regressions.^17^

In Table 1 we provide summary statistics for our analysis samples, stratified by survey year. Observations in the Table are children, corresponding to the level of analysis in the regressions below.^18^ Haemoglobin data were collected for all children 6–59 months old in the 2006 survey but only for a random subsample of 50% of children in this age range in the 2011 survey. Within the main analysis sample (*N* = 6,464) that contains haemoglobin data and the main control set, dietary information was collected for a smaller subsample (*N* = 4,348) that targeted the younger children in each household. Similarly, information on mother's use of iron supplementation during pregnancy was only recorded with respect to the most recent pregnancy for each mother, leading to a somewhat smaller sample size (*N* = 4,720) relative to the main analysis sample. Table 1 presents summary statastics for the main (‘full’) sample and for the dietary module (‘food’) subsample. For completeness, Table A2 repeats the summary statistics for the subsample of children for which we observe whether the mother took iron during pregnancy.

**Table 1:** Summary Statistics

	Full sample				Food subsample			
	2006 Round		2011 Round		2006 Round		2011 Round	
	Mean	SD	Mean	SD	Mean	SD	Mean	SD
	(1)	(2)	(3)	(4)	(5)	(6)	(7)	(8)
Child haemoglobin (g/dL)	11.16	1.44	11.21	1.41	10.99	1.45	10.86	1.38
Child anaemia, mild (<11 g/dL)	0.42	0.49	0.41	0.49	0.47	0.50	0.51	0.50
Child anaemia, moderate (< 10 g/dL)	0.19	0.39	0.17	0.38	0.23	0.42	0.24	0.43
Child anaemia, severe (< 7 g/dL)	0.01	0.07	0.00	0.07	0.01	0.09	0.01	0.09
Child age in months	33.10	15.44	32.94	15.35	28.54	14.67	24.56	15.24
Child male	0.50	0.50	0.52	0.50	0.48	0.50	0.47	0.50
Household open defaecation	0.59	0.49	0.46	0.50	0.61	0.49	0.50	0.50
Household sanitation (1 ‐ open defaecation)	0.41	0.49	0.54	0.50	0.39	0.49	0.50	0.50
Household electricity	0.40	0.49	0.66	0.47	0.37	0.48	0.61	0.49
Household improved water source	0.78	0.41	0.87	0.34	0.77	0.42	0.85	0.35
Household has health card	0.22	0.42	0.26	0.44	0.26	0.44	0.34	0.47
Household urban	0.21	0.41	0.20	0.40	0.20	0.40	0.18	0.38
Mother some education	0.38	0.48	0.52	0.50	0.37	0.48	0.52	0.50
Mother literate	0.46	0.50	0.59	0.49	0.45	0.50	0.58	0.49
Child took parasite medication, last 6 months	0.75	0.43	0.77	0.42	0.71	0.46	0.66	0.47
Mother took iron supplements during pregnancy*	0.53	0.50	0.78	0.42	0.56	0.50	0.82	0.38
Child ate fruits and vegetables, last 24 hours					0.60	0.49	0.40	0.49
Child ate meat and eggs, last 24 hours					0.21	0.41	0.23	0.42
Count of kinds of food child ate, last 24 hours					2.29	1.23	1.97	1.25
Observations	4,469		1,995		3,371		997	

*Notes*. The Table displays summary statistics for the 2006 and 2011 rounds of the Nepal Demographic and Health Survey (DHS). Statistics are calculated separately for our main analysis sample (‘full sample’) and for the subsample in which dietary information was collected (‘food subsample’). Observations are children aged 6–59 months. The sample sizes are smaller in 2011 than 2006 by survey design. In 2006, the DHS collected haemoglobin data from all children aged 6–59 months but in 2011 collected haemoglobin data for a random 50% subsample in this age range. *Information on mother's use of iron supplementation during pregnancy was only recorded with respect to the most recent pregnancy for each mother. See Table A2 for an additional set of summary statistics calculated over the subsample for which the iron supplementation question was asked.

Table 1 shows that the use of intestinal parasite medicine was fairly stable over the 2006–11 period. Over the same period, there was a large decline in open defaecation. Toilet and latrine use (one minus open defaecation) climbed by an average of 13 percentage points nationally. Children's consumption of meat and eggs was unchanged over time, though fruit and vegetable consumption declined nationally, suggesting a relative deficit in some nutrients critical to haemoglobin production. Dietary diversity, defined as the number of kinds of foods (meat and eggs, plant protein, fruits and vegetables, starches, and dairy) consumed, also declined. This worsening of food intake, trending opposite to the improved sanitation, may account for the essentially unchanged average level of haemoglobin between the 2006 and 2011 survey rounds, though the stable national average masks significant regional variation over time. In terms of general economic and infrastructure development, household electrification and access to improved water sources also significantly increased over this period.

### 3.2 Identifying Variation

**Figure 2:**
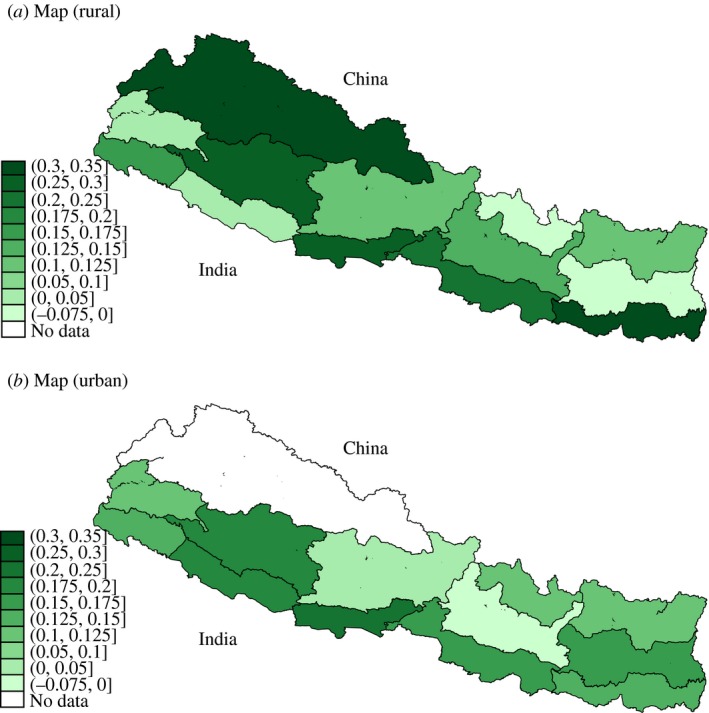
Identifying Variation: Within‐region Sanitation Improvements, 2006–11. (a) Rural Areas of 13 Regions of Nepal. (b) Urban Areas of 12 Regions of Nepal *Notes*. The Figure shows improvements in sanitation between the 2006 and 2011 rounds of the Nepal DHS. Regions are colour‐coded according to the change in fraction of households using toilets and latrines (equal to one minus the fraction of households defaecating in the open). Darker greyscale description indicate lesser improvements. Panel (*a*) includes only rural households within each of 13 regions. Panel (*b*) includes only urban households of 12 regions. In panel (*b*), the Western Mountain Region (upper left) contained no urban areas. Colour figure can be viewed at http://wileyonlinelibrary.com

Between the 2006 and 2011 rounds of the Nepal DHS there was a large reduction in open defaecation. Importantly for our study, this improvement varied significantly across the 25 regions described above. To illustrate the difference‐in‐differences variation that we exploit for identification, Figure 2 shows how open defaecation changed within regions over time. Panel (*a*) of Figure 2 plots the improvement in sanitation (one minus the mean open defaecation rate) for rural areas. Panel (*b*) repeats the plot for urban areas. Shading correspond to the sanitation improvement, with greyscale description indicating more improvement.

Regional changes in the fraction of households practicing open defaecation (${\bar{OD}}_{r}$) ranged from as little as zero to more than a 30 percentage point reduction in just 5 years. To put the size of this decline in context: in India, which has similarly poor sanitation and has experienced fast economic growth over the last two decades, reduced open defaecation by only about 1 percentage point per year between 2001 and 2011 (Government of India, 2011).

While it is not the goal of this article to evaluate any particular policy – or even to catalogue the determinants of open defaecation in Nepal over our study period – two facts are relevant to understanding the panel variation we exploit. First, beginning in the mid‐2000s, policy attention by Nepal's central government turned more significantly towards sanitation. It is likely that this policy priority change and the resulting investment both drove changes in demand for latrines among Nepalis and was also reflecting changing national attitudes. Second, the effective ‘exposure’ of a locality to government‐led sanitation activities at the national level was heavily constrained by the current level of sanitation development in the locality. Places with historically worse sanitation had a larger scope for improvement in level terms.

The relevant policy changes originated at the national level in Nepal's Department of Water Supply and Sewerage, which in 2004 began launching initiatives aimed at improving sanitation. New National Guidelines for Hygiene and Sanitation Promotion were announced in 2005. At the time, Nepal's government had made a commitment to decentralising the administration of government functions. In accordance with that broader policy commitment across areas of government, responsibility for determining the details of implementing sanitation policy rested with individual Village Development Committees and District Development Committees.^19^ Though there is no census of the local programmes developed during this timeframe, activities included various subsidy programmmes for toilet construction as well as marketing campaigns aimed at increasing demand for toilets and latrines.^20^

With respect to an area's ‘exposure’ to national changes, it is plausible that national resources devoted towards curbing open defaecation had greater scope for impact in places where the problem was more severe. In the extreme, this was certainly true: in the case of zero open defaecation at baseline (true for one urban region), there was no room to improve further and there was consequently no significant change in open defaecation over the 2006–11 period. In contrast, in regions with the worst sanitation in 2006 (with open defaecation rates as high as 70%), open defaecation levels declined by around 30 percentage points. This type of variation is similar to that used in a wide range of studies that identify impacts of national programmes by generating a potential exposure measure that varies across geography (Finkelstein, 2007).^21^ In a study related to ours, Bleakley (2007) exploited geographic variation in the pre‐treatment hookworm infection rate in regions of the US South to identify effects of a deworming campaign on human capital outcomes. While the hookworm campaign ‘treatment’ was similar across areas, effective exposure was greater where the pre‐programme infection rate was higher.

**Figure 3:**
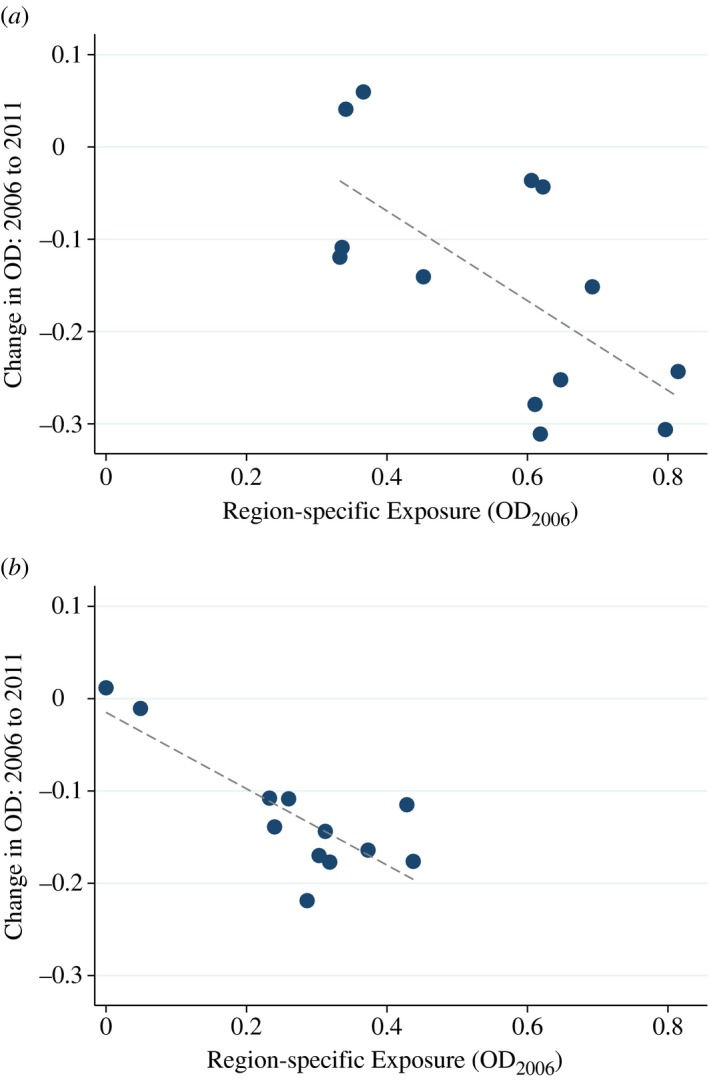
Baseline Sanitation in 2006 Predicts Subsequent Improvement. (a) Rural Areas of 13 Regions. (b) Urban Areas of 12 Regions *Notes*. The Figure plots the relationship between a region's *Exposure* to national sanitation improvements and the subsequent within‐region change in open defaecation (OD) between 2006 and 2011. *Exposure* is defined as the baseline level of open defaecation in 2006. Panel (*a*) includes only rural households within each of 13 regions. Panel (*b*) includes only urban households. The number of regions in panel (*b*) is smaller because the Western Mountain Region of Nepal contained no urban areas. Regressions estimating the slopes are presented in Table A3. Slopes are significant at p < 0.01. Colour figure can be viewed at http://wileyonlinelibrary.com

Figure 3 demonstrates the idea in our setting, plotting the change in regional open defaecation from 2006 to 2011 on the vertical axes against the 2006 level along the horizontal axes. Rural regions are plotted as scatter points in panel (*a*) and urban regions are in panel (*b*). Sanitation improved essentially everywhere – in 22 out of the 24 regions with non‐zero baseline open defaecation. But both panels show that such reductions were larger in regions with higher levels of open defaecation in 2006. Regressions corresponding to the figure in Table A3 confirm the statistical significance of the relationship at p < 0.01. In fact, the estimated slopes in the two panels are numerically identical at −0.45. Therefore, without discounting the notion that the details of local policies mattered in many ways, we note that the regional variation in improvement between the survey rounds appears to be strongly determined by the baseline level of sanitation in 2006.^22^

Given the standard parallel trends assumption underlying our difference‐in‐differences analysis, it is important for us to demonstrate that the places with greater scope for sanitation improvement (i.e. greater ‘exposure’ to the overall national sanitation improvement trend) were not differentially trending along other variables that might independently affect haemoglobin. Because the previous medical science points to nutrition, iron supplementation and deworming as the known determinants of low haemoglobin, non‐parallel trends in any of these three health inputs would be of particular concern. Fortunately, the DHS data can be used to provide direct evidence on each of these, and we investigate below whether patterns in these other health inputs where trending differentially in places that experienced the greatest improvements in sanitation. Here, we begin by noting that Figure 2 shows that improvements were not limited to low‐lying regions (on the southern, Indian border) or mountainous regions (on the northern, Chinese border). Nor were improvements concentrated in contiguous areas of the country. Interestingly, there also appears to be no positive association between improvements in the rural and urban areas of the same regions: the pattern of improvement in rural places (panel (*a*)) is not paralleled in the urban places (panel (*b*)) of the same geographic areas.

### 3.3 Empirical Framework

We organise our analysis at the level of the child, including as observations all children for whom haemoglobin was measured (children aged six months to five years). We regress haemoglobin concentration (*Hb*) on the mean open defaecation at the regional level (${\bar{OD}}_{rt}$):

(1) $$Hb_{irt}=\beta_{1}{\bar{OD}}_{rt}+\beta_{2}OD_{irt}+\alpha_{2011}+\sum_{r\in R}\alpha_{r}+f\left(X_{irt}\right)+\epsilon_{irt}.$$

Here, *i* indexes children, *r* indexes the regions over which we construct our panel, and *t* indexes the DHS two survey rounds. The dependent variable $Hb_{iprt}$ is a continuous variable for blood haemoglobin concentration. $OD_{iprt}$ controls for whether the child's own household defaecates in the open. This ensures that we are identifying external effects of local sanitation. We additionally control for a variety of person, household and region‐level characteristics in $f(X_{iprt})$ to demonstrate robustness. These are described in more detail below. Fixed effects for survey round ($\alpha_{t}$) and fixed effects for each of 25 regions ($\alpha_{r}$) control for all common time trends and any place‐specific characteristics that could otherwise confound results. The coefficient of interest, $\beta_{1}$, identifies the impact of a change in the mean of regional open defaecation on childrens’ haemoglobin levels.

In (1), we have aggregated the sanitation environment measure (*OD*) up to the level of the region so that we can exploit region fixed effects. The alternative approach of disaggregating the locality data further and including neighbourhood fixed effects is not possible here because the DHS sampling scheme did not re‐interview in the same neighbourhoods in the 2006 and 2011 rounds.

## 4 Results

### 4.1 Main Findings

We present the main regression results in Table 2, where the dependent variable across all columns is haemoglobin in children aged 6–59 months. The negative coefficient estimates in Table 2 show that improvements in sanitation – i.e. lower rates of open defaecation – are associated with higher concentrations of haemoglobin in children. Point estimates are larger in the difference‐in‐differences than in the cross‐sectional OLS regressions, though not statistically different.^23^

**Table 2:** Main Results: Effects of Open Defaecation (OD) on Children's Haemoglobin

Dependent variable:	Haemoglobin (g/dL)					
Specification:	Pooled cross‐section			D‐in‐D		
Sample:	Full	Full	Full	Full	Full	Full
	(1)	(2)	(3)	(4)	(5)	(6)
Region mean OD	−0.91^*^	−0.59^*^	−0.90^*^	−1.53^*^	−1.19^*^	−1.27^*^
	(0.17)	(0.18)	(0.25)	(0.49)	(0.48)	(0.50)
Survey round FEs	X	X	X	X	X	X
Age‐in‐month × sex indicators	X	X	X	X	X	X
Own OD		X	X		X	X
Economic controls			X			X
Mother education controls			X			X
Parasite medicine			X			X
Time‐varying region controls			X			X
Region FEs				X	X	X
Mean of dependent variable	11.2	11.2	11.2	11.2	11.2	11.2
Observations	6,464	6,464	6,464	6,464	6,464	6,464

*Notes*. The Table reports results from a series of OLS regressions. The dependent variable in all columns is the child's haemoglobin level. All columns control for survey round fixed effects, religion indicators, and a complete set of age‐in‐month × sex indicators. Columns (4)–(6) include region fixed effects. Economic controls include indicators for five household assets asked about in both survey rounds, an indicator for household electricity and indicators for type of wall materials (9 categories), roof materials (8 categories), and floor materials (6 categories) that makeup the child's dwelling. Parasite medicine is an indicator for the consumption of intestinal parasite medication in the last six months. Mother's education controls include an indicator for literacy and indicators for levels of educational attainment (4 categories). Region controls add continuous measures of electrification and the use of improved water sources at the regional level. Observations are children. Standard errors are clustered at the PSU level. +p < 0.1, *p < 0.05, **p < 0.01.

Columns (1)–(3) estimate the coefficient on ${\bar{OD}}_{rt}$ from (1) using pooled cross sectional data. Columns (4)–(6) add fixed effects for the 25 regions defined by geographic area interacted with urban. Adding region fixed effects changes these OLS regressions from a pooled cross sectional analysis to a difference‐in‐differences analysis, identified within regions over time. Regions with smaller or no improvements in sanitation implicitly serve to control for common time trends.

All columns control for survey round fixed effects, region indicators and a complete set of 108 age‐in‐month × sex indicators to flexibly adjust for any biological differences in haemoglobin by age and sex. The control for ‘own OD’ indicates whether the child's own household practices open defaecation.^24^ Economic controls include indicators for five household assets asked about in both survey rounds, an indicator for household electricity and controls for the quality of the child's dwelling, including the materials used in construction of the roof, walls and floor.^25^ Mother's education controls include an indicator for literacy and indicators for levels of educational attainment (4 categories). ‘Parasite medicine’ is an indicator for the child's consumption of intestinal parasite (deworming) medication within the last six months. Regional controls add time‐varying region characteristics that are not absorbed by the regional fixed effects. These include mean household electrification and mean household access to protected/improved water sources within each region × year.^26^ The inclusion of economic controls, mother characteristics, medicine controls and time‐varying region‐level controls has only minor effects on point estimates, indicating that our regressors of interest are not strongly correlated with these variables.

**Table 3:** Robustness: Instrumenting within‐Region Sanitation Changes with 2006 Exposure

Dependent variable:	Change in mean haemoglobin (g/dL) within region			
Specification:	OLS	OLS	IV	IV
	(1)	(2)	(3)	(4)
Change in mean OD within region	−1.53*	−1.72**	−2.86*	−2.88**
	(0.55)	(0.54)	(1.44)	(0.71)
Urban		X		X
Change in mean electrification within region		X		X
Change in mean improved water source within region		X		X
Mean of dependent variable	−0.10	−0.10	−0.10	−0.10
First stage F‐stat			16.3	25.3
Observations (regions)	25	25	25	25

*Notes*. The Table reports results from a series of OLS and IV regressions in which the unit of analysis is the region. The dependent variable in all columns is the change in regional mean of children's haemoglobin levels between 2006 and 2011. IV regressions in columns (3) and (4) instrument for the change in mean open defaecation within the region ($\Delta {\bar{OD}}_{r}$) with an exposure variable, defined as the 2006 level (${\bar{OD}}_{r,2006}$). First stage regressions corresponding to columns (3) and (4) are displayed in Table A3. Observations are the 25 regions, defined by 13 geographic areas interacted with an urban indicator. Robust standard errors are reported. +p < 0.1, *p < 0.05, **p < 0.01.

The mean reduction in open defaecation nationally was on the order of a 10 percentage point decline. A coefficient of −1.27 on neighbourhood level OD (column (6)) indicates that a 10 percentage point reduction in the fraction of neighbours defaecating in the open yields an improvement in haemoglobin of 0.127 g/dL, or about 9% of a standard deviation. To put this effect size in context, interventions such as daily micronutrient supplementation (Friis *et al*., 2003), iron supplementation (Lind *et al*., 2003), fortification (Van Stuijvenberg *et al*., 1999) and treating for parasites (Friis *et al*., 2003; Taylor‐Robinson *et al*., 2012) have effect sizes that range from 0.20 to 0.40 g/dL.

Thus, the sanitation‐haemoglobin effect is large, though not implausibly so. Recall that one of the two channels by which we hypothesise poor sanitation may impact haemoglobin is via intestinal worms, which are spread by skin contact with human faecal matter. In a double‐blind randomised control trial in Kenya, Friis *et al*. (2003) find that a single dose of deworming medicine generated an increase in haemoglobin of 0.21 g/dL measured eight months after its administration, an effect similar in size to that from an alternative treatment arm of the same study that administered daily supplements of 13 micronutrients (including iron) for eight months.

Standard errors in parentheses are clustered at the PSU‐level across all columns. Because the difference‐in‐differences analysis uses variation in mean open defaecation at the level of the region‐by‐year, we also investigate alternative clustering at the region‐by‐year level. The exercise, which requires a special asymptotic refinement due to the small number of clusters (Cameron *et al*., 2008; Cameron and Miller, 2015), is described in Appendix A.3. Results from several alternative clustering schemes are tabulated in Table A4, which shows that the statistical significance of the results is robust to even the most aggregated level of clustering (25 regions) and even after performing the proper asymptotic refinement (via wild cluster bootstrapping).

For completeness, in Table A5 we report on alternative specifications that re‐estimate our main results in Table 2, using various thresholds for anaemia as the dependent variables rather than the continuous haemoglobin measure. The anaemia thresholds that constitute the dependent variables correspond to the World Health Organization standards for mild (<11 g/dL), moderate (<10 g/dL), and severe (<7 g/dL) anaemia. The binary outcome measures generate relatively large confidence intervals,^27^ though for anaemia defined as moderate or worse, effects are statistically significant at the 5% level in both the cross‐sectional and difference‐in‐differences specifications.

Finally, for robustness, we also report on an alternative IV estimation strategy in Table 3 that aggregates up to the level of the region and exploits the variation displayed in Figure 3, in which regions with worse starting points had greater scope for rapid improvement. For these regressions, we define an exposure variable equal to the 2006 level of open defaecation. We aggregate all data to the region and take first differences from 2006 to 2011 for our dependent and independent variables of interest, denoting changes with Δ. This results in 25 observations. We run the following regression, which instruments the within‐region sanitation change, ${\Delta \bar{OD}}_{r}$, with region‐specific exposure to national changes:

(2) $$\Delta {\bar{Hb}}_{r}=\beta_{1}{\hat{\Delta \bar{OD}}}_{r}+\epsilon_{r},$$

(3) $${\hat{\Delta \bar{OD}}}_{r}=\theta Exposure_{r}+\mu_{r}.$$

This specification has the advantage of identifying estimates using only variation in sanitation improvement over time that is predicted by the 2006 level within the region. This is the variation depicted in Figure 3.^28^ An additional advantage of the regression in (2) is that it is not subject to the small number of clusters issue discussed above. Robust standard errors are consistent here without asymptotic refinement.^29^ Of course, this method has the drawback of not allowing inclusion of individual‐level covariates and of estimating the coefficient of interest off of just 25 observations. Nonetheless, the results in Table 3 align closely with the main results in Table 2.

In the context of this regression, it is also important to note that the exposure variable is not merely capturing a mean reversion pattern. Mean reversion would occur if regions experienced a simultaneous negative shock to both children's haemoglobin and sanitation prior to the 2006 DHS survey round and then rebounded by the 2011 round. In contrast to this type of pattern, changes in sanitation tend to be unidirectional (towards improvement). We verify the unidirectional nature of sanitation changes in our setting using an earlier 2001 round of the Nepal DHS. This earlier round contains data on open defaecation for 23 of our 25 regions but pre‐dates the measurement of haemoglobin by the DHS. Sanitation improved within most regions (76%) between 2001 and our base year, 2006, just as it did between 2006 and 2011 (in 88% of regions), which is inconsistent with mean reversion. In the few regions where sanitation worsened from 2001 to 2006, the change was relatively small. In addition, there was no significant correlation between the 2001/2006 change in open defaecation ($\Delta {\bar{OD}}_{r,2000/2006}$) and the 2006/2011 change ($\Delta {\bar{OD}}_{r,2006/2011}$), contrary to mean reversion. Nor was there a significant correlation between indicators for the signs of these changes – i.e. a region experiencing a sanitation decline from 2001 to 2006 was not more likely to experience a sanitation improvement from 2006 to 2011. Similarly, creating two rankings of regions according to their 2001/2006 changes and their 2006/2011 changes and performing the Spearman's test for rank correlation did not reject the null of independence between the earlier and later within‐region sanitation changes.

### 4.2 Parallel Trends Tests

In this subsection, we evaluate the robustness of our results to alternative hypotheses. We begin by examining whether the sanitation improvements we exploit were correlated with other changes in children's health inputs. Put differently, we investigate whether the parallel trends assumption central to our difference‐in‐differences strategy holds for the variables that we can observe. The most well‐established cause of anaemia in children is poor diet and, in particular, a lack of dietary iron. Deworming medications have also been proved to be important. If sanitation improvements were correlated with improvements in diet, iron intake, and deworming medicines, it would provide evidence against our identification strategy. The DHS allows us to observe data on each of these inputs directly.^30^

To test for parallel trends in observables, in Table 4, we repeat the regression analysis in Table 2 but substitute alternative dependent variables in place of haemoglobin. These include three dietary variables in columns (1)–(6): whether the child ate fruits and vegetables in the last 24 hours; whether the child ate meat and eggs in the last 24 hours; and the variety of diet in the last 24 hours, operationalised as a count of food types (starches, plant protein, fruits and vegetables, meat and eggs, and dairy). Columns (7)–(10) repeat this trends test for an indicator of whether the child took deworming medications in the last six months and an indicator for whether the mother took iron supplements during pregnancy.^31^

**Table 4:** Parallel Trends Assumption: Sanitation Improvements Uncorrelated with Changes to Diet and Medicine‐taking

Dependent variable:	Child ate meat and eggs, last 24 hours		Child ate fruits and vegetables, last 24 hours		Dietary diversity: diff. kinds of food, last 24 hours		Child intestinal parasite medicine (deworming)		Mother iron supplements during pregnancy	
Specification:	Cross‐section	D‐in‐D	Cross‐section	D‐in‐D	Cross‐section	D‐in‐D	Cross‐section	D‐in‐D	Cross‐section	D‐in‐D
Sample:	Food	Food	Food	Food	Food	Food	Full	Full	Iron	Iron
	(1)	(2)	(3)	(4)	(5)	(6)	(7)	(8)	(9)	(10)
Region mean OD	−0.07	0.14	−0.08	0.11	−0.36+	0.55	0.10	0.21+	0.02	−0.15
	(0.09)	(0.20)	(0.10)	(0.21)	(0.21)	(0.46)	(0.06)	(0.13)	(0.09)	(0.17)
Survey round FEs	X	X	X	X	X	X	X	X	X	X
Age‐in‐month × sex indicators	X	X	X	X	X	X	X	X	X	X
Own OD	X	X	X	X	X	X	X	X	X	X
Economic controls	X	X	X	X	X	X	X	X	X	X
Mother education controls	X	X	X	X	X	X	X	X	X	X
Parasite medicine	X	X	X	X	X	X			X	X
Time‐varying region controls	X	X	X	X	X	X	X	X	X	X
Region FEs		X		X		X		X		X
Mean of dependent variable	0.21	0.21	0.56	0.56	2.22	2.22	0.76	0.76	0.61	0.61
Observations	4,348	4,348	4,348	4,348	4,348	4,348	6,464	6,464	4,720	4,720

*Notes*. The Table reports results from a series of OLS regressions that test the parallel trends assumption. The dependent variables in the Table differ across columns. Each dependent variable represents an outcome or behaviour which is likely to affect haemoglobin levels directly. These dependent variables include an indicator for whether the child has eaten meat or eggs in the last 24 hours, an indicator whether the child has eaten fruits or vegetables in the last 24 hours, a continuous variable for the count of kinds of different foods the child has eaten within the last 24 hours, an indicator for whether the child has taken intestinal parasite medication within the last six months, and an indicator for whether the mother took iron supplements during pregnancy. Controls are as described in the Table 2 notes. Observations are children. Sample sizes vary because survey information on diet was collected for only a subsample of children in each household (*N* = 4,348) and because survey information about maternal iron supplementation was only collected for each mother's most recent pregnancy (N = 4,720). See Table A2 and text for full details. Standard errors are clustered at the PSU level. +p < 0.1, *p < 0.05, **p < 0.01.

Specifications in Table 4 are similar to those in Table 2. For each of the five dependent variables, haemoglobin would be expected to be increasing in the variable's levels based on the prior literature. The Table shows no evidence that improving sanitation was correlated with improvements in diet or increases in iron supplementation or deworming. Across the columns, the correlations with sanitation improvements are not statistically significant. Further, the small point estimates are positive, which if anything would bias against our estimates. Positive point estimates indicate that in places were sanitation was improving more quickly (i.e. greater relative decline in open defaecation), diet and deworming were improving less (or declining more). If our effects were due spurious correlations between changes in sanitation and changes in diet, deworming, or iron supplementation, then one would expect negative estimates here, showing that reductions in open defaecation were correlated with relative increases in these variables.

Leaving aside the significance of the estimates in Table 4, positive effects of sanitation on food consumption and medicine taking could be consistent with a behavioural response to the biological mechanism we claim to identify. In principle, if parents observed less lethargy and weakness in their children due to improvements in sanitation (and its effects on haemoglobin), they may have endogenously responded to lower rates of open defaecation by scaling back intestinal parasite medicine.^32^ Similarly, if children faced less diarrhoeal disease due to the improving sanitation environment, parents may have endogenously responded by reducing the child's food consumption, as a greater fraction of calories consumed would be absorbed and retained in the better disease environment. This could lead to positive coefficient estimates in the Table. Our estimation strategy implicitly captures any such general equilibrium effects, in which some of the haemoglobin gains of improved sanitation are counteracted by the compensating reductions in other health inputs. From the perspective of the narrower question of the impacts of sanitation on haemoglobin holding all other behaviour fixed, it would imply that our estimates are underestimates.

In summary, the results from the Table 4 analyses suggest that the relationship between open defaecation and children's haemoglobin is not merely due to differential trends in ‘treated’ regions along other known determinants of haemoglobin levels. It is important to keep in mind that diet, micronutrient supplementation, malaria, and deworming treatments are the only factors that have been shown to affect of anaemia in the prior medical, epidemiological, or economic literature. Since malarial incidence is very low in our country context, the results in Table 4 directly confront this short list of known causal factors, and therefore provide evidence that we have identified a new contributing factor to anaemia.

As an additional robustness check, we also replicate the main results in Table 2 with the inclusion of the diet controls. Dietary data were not collected for about a third of our main estimation sample and adding these controls reduces precision in the haemoglobin‐sanitation regressions. Nonetheless, Table A6 shows that adding dietary controls leaves point estimates essentially unchanged. These new controls include indicators for the child's consumption of meat and eggs in the last 24 hours and fruits and vegetables in the last 24 hours, as well as a control for the number of different kinds of foods in the last 24 hours and indicators for the counts of meals consumed over the last 24 hours.^33^

We next report on a series of tests that evaluate whether improvements in other development indicators (with no obvious theoretical link to anaemia) predict changes in haemoglobin over the panel. The aim is to evaluate whether sanitation improvements were merely part of a broader package of local improvements and whether such broader trends in local development caused haemoglobin changes or were potentially correlated with an unobservable determinant of haemoglobin.^34^ If within‐region, across‐time changes in other markers for local development predicted haemoglobin changes, it would not be an identification problem *per se* but it would suggest the need for care in separating the effects of sanitation improvements from other development indicators.

The summary statistics reveal that nationally, household electrification increased by 26 percentage points from 2006 to 2011 and household access to ‘improved’ water sources increased by 9 percentage points. We investigate these infrastructure variables, along with a measure of the social safety net: whether the household has a national health card. The latter measure improved by 4 percentage points (18%) from 2006 to 2011.

The pattern of the main results in Table 2 already provides some evidence against the notion that sanitation improvements were merely a marker for a wider package of improvements with independent effects on haemoglobin. For example, a comparison of columns (5)–(6) in Table 2 reveals that the inclusion of controls for time varying regional measures of electrification and access to improved water sources had essentially no effect on the estimated effects of ${\bar{OD}}_{rt}$ and ${\bar{OD}}_{prt}$ on haemoglobin.^35^ This indicates that these improvements were either uncorrelated with improvements in open defaecation or uncorrelated with changes in children's haemoglobin levels. Table A7 provides direct evidence in support of the parallel trends assumption, showing that there is no statistically significant association between changes in sanitation and changes in electrification or water access within regions.

In Table 5, we take an alternative approach to examining the possibility of confounding trends by replicating the exact analysis in Table 2 but substituting measures of electrification, water infrastructure and health card possession in place of open defaecation as the regressors of interest. Specifications in Table 5 mirror those in columns (3) and (6) of Table 2 and the corresponding regressions from the main results are repeated in the last two columns for reference. Each regressor is constructed as a bad (e.g. the lack of electricity) so that signs of coefficients are more easily comparable to those for the open defaecation variable, which is also a bad. The Table shows that with respect to electrification, access to improved water sources, and health card possession, there is no consistent relationship between changes in these variables and haemoglobin outcomes.^36^

**Table 5:** Other Regional Improvements Do Not Predict Haemoglobin Changes

Dependent variable:	Haemoglobin (g/dL)							
Regressor of interest:	No electricity		No improved water source		No health card		OD effects (repeated for reference)	
Specification:	Cross‐section	D‐in‐D	Cross‐section	D‐in‐D	Cross‐section	D‐in‐D	Cross‐section	D‐in‐D
Sample:	Full	Full	Full	Full	Full	Full	Full	Full
	(1)	(2)	(3)	(4)	(5)	(6)	(7)	(8)
Region mean (lacking electrification)	0.28+	0.21						
	(0.15)	(0.31)						
Region mean (unimproved water source)			1.56**	0.54				
			(0.25)	(0.39)				
Region mean (family lacks healthcare card)					0.21	0.19		
					(0.31)	(0.72)		
Region mean (open defaecation)							−0.90**	−1.27*
							(0.25)	(0.50)
Survey round FEs	X	X	X	X	X	X	X	X
Age‐in‐month × sex indicators	X	X	X	X	X	X	X	X
Own [electrification/water/card]	X	X	X	X	X	X	X	X
Economic controls	X	X	X	X	X	X	X	X
Mother education controls	X	X	X	X	X	X	X	X
Parasite medicine	X	X	X	X	X	X	X	X
Time‐varying region controls							X	X
Region FEs		X		X		X		X
Mean of dependent variable	11.2	11.2	11.2	11.2	11.2	11.2	11.2	11.2
Observations	6,464	6,464	6,464	6,464	6,464	6,464	6,464	6,464

*Notes*. The Table reports results from a series of OLS regressions. The dependent variable in all columns is the child's haemoglobin level. The regressor of interest varies across columns. In columns (1) and (2), the regressor of interest is the regional mean of an indicator for households lacking electricity. In columns (3) and (4), it is the regional mean of an indicator for households lacking access to ‘improved’ water sources, which include piped water, protected wells and protected springs. In columns (5) and (6), the regressor of interest is the regional mean of an indicator for households lacking a national health card. Controls are as described in the Table 2 notes. Observations are children. Standard errors are clustered at the PSU level. +p < 0.1, *p < 0.05, **p < 0.01.

In all cases, these tests support the identifying assumption that the changes in local open defaecation we study were not merely markers for broader local development changes that had independent and confounding effects on our outcome of interest. None of the difference‐in‐differences results are significant and, moreover, the positive signs of the point estimates in Table 5 are opposite to what would be expected if local trends in these variables were reflecting an omitted factor: a positive sign indicates that where these deficiencies (e.g. lacking electricity) were increasing (or decreasing by less), haemoglobin was differentially improving. This is in contrast to the theoretically grounded and statistically significant negative effects in the case of open defaecation.

## 5 Discussion and Policy Implications

Today, about 14% of the world's population practices open defaecation. Given the scope of this practice, our estimates imply that poor sanitation could play an important role in explaining variation in anaemia rates worldwide. Indeed, our IV estimates – identified within Nepali localities over time – are of the same order of magnitude as the cross‐country correlations displayed in Figure 1.^37^

The hypothesis that sanitation has economically important impacts on haemoglobin and anaemia is new but it fits together with several pieces of recent evidence from the economics literature (in addition to the evidence from the epidemiological and medical literature presented in Section 1). Several randomised trials have found an effect of sanitation programmes on child height (Cameron *et al*., 2013; Gertler *et al*., 2015; Hammer and Spears, 2016). The biological mechanisms linking open defaecation and height are similar to the proposed link between open defaecation and haemoglobin status, supporting the plausibility of our findings. Nonetheless, there have been no randomised controlled trials of the effect of latrine or toilet provision on haemoglobin status. Our study is the first to establish this link.^38^ Our findings on anaemia may also help to explain some of the recent empirical results in the economics literature. Spears and Lamba (2016) find that exposure to open defaecation is associated with lower child cognitive function. Such effects would be consistent on the wider literature about the impacts of anaemia on cognition (Stoltzfus *et al*., 1997).

Our findings have two key implications for researchers and policy makers interested in anaemia. First, with respect to research, our study demonstrates the need for future work on anaemia and sanitation but it also suggests that any randomised trial that implements an intervention targeting anaemia at the individual level will necessarily miss an important phenomenon. This is because the phenomenon we study is related to the behaviour of neighbours: neighbours? open defaecation introduces germs and parasites into the child's body. Therefore, changing the open defaecation practice of an individual household or deworming individual children (or otherwise arresting the process by which faecal pathogens disrupt the absorption of critical nutrients) are likely to yield very different impacts on haemoglobin than those interventions that randomise at the neighbourhood level. This point about the public goods nature of sanitation has been made in the context of deworming interventions by Miguel and Kremer (2004), Bundy *et al*. (2009) and others. Because open defaecation primarily constitutes an externality, research based on individual randomization cannot uncover it.

A related point with respect to policy is that the public goods nature of sanitation also suggests a new set of tools available to address anaemia that are fundamentally different from the *status quo*, which generally involves administering an iron supplement, fortified food, or a deworming pill to an individual child. Our findings imply that policy action can be taken at a community level and that the reduction of anaemia in children may even require action on the part of people who are neither parents nor children. This substantially expands the set of policy responses available for targeting anaemia.

Acknowledging that open defaecation has external effects on anaemia will require a significant shift in thinking for many researchers and policy makers, who tend to consider anaemia and other nutritional diseases to be problems of inadequate food intake, and to overlook the important role of disease in determining ‘nutritional’ outcomes. Recommendations and interventions aimed at anaemia by leading development organisations almost always focus on food‐intake based interventions, either in the forms of iron, vitamin B12 and folate supplementation and fortification, or through efforts to encourage people to diversify their diets. However, since exposure to poor sanitation can lead to nutritional loss to intestinal parasites and to the malabsorption of critical nutrients (via enteropathy), sanitation plays a critical role in determining net nutrition.

We do not mean to claim that our findings offer a pathway for a simple solution to the global problem of anaemia. Changing behaviour with respect to open defaecation has proven very difficult in many settings. Coffey *et al*. (2014) catalogues how (stated and revealed) preferences for open defaecation can be deeply rooted and not merely a matter of the affordability of toilets. The low private demand for latrines and toilets may be owing to inaccurate beliefs with respect to the private benefits, as well as coordination problems and the classic problem of under‐investment in goods with external benefits. Nonetheless, our results offer new evidence of such benefits, and in this way strengthen the basic economic rationale for policy intervention.

## 6 Conclusion

Our study is the first to empirically investigate the hypothesis that poor sanitation is an important contributor to low haemoglobin and anaemia in children. The results here suggest new policy avenues for addressing anaemia in the developing world, as the elimination of open defaecation is rarely among priority policy recommendations or the focus of programmes implemented to fight anaemia. The finding that open defaecation significantly impacts these outcomes adds to a rapidly expanding literature on the importance of open defaecation in shaping human capital outcomes. More broadly, our findings connect to a wide literature on the role of water, sanitation and the disease environment in driving health and human capital accumulation in the developing world and the historical United States.

## Supporting information


**Data S1.**

## Figures and Tables

**Figure A1:**
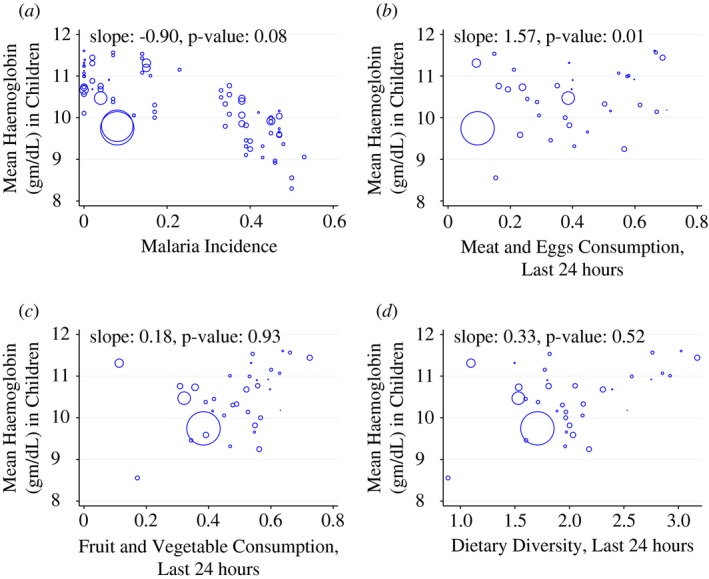
Cross‐country Relationships: Haemoglobin *Versus* Diet and Malaria. (a) Malaria. (b) Meat and Eggs. (c) Fruits and Vegetables. (d) Dietary Diversity *Notes*. The Figure shows the cross‐country relationship between diet variables and malaria infection rates and haemoglobin levels in children aged 6–35 months. Each point in the scatterplots corresponds to a country × year mean of a Demographic and Health Survey. Marker sizes are proportional to country populations. The 81 DHS surveys represented in panel (*a*) are listed in Table A1. For panels (*b*)– (*d*), only the 35 DHS surveys that collected information on the food consumption variables are included. In panel (*b*) the horizontal axis measures the fraction of children consuming meat or eggs in the 24 hours before the survey. In panel (*c*) the horizontal axis measures the fraction of children consuming fruit/vegetables in the 24 hours before the survey. In panel (*d*) the horizontal axis measures the number of different kinds of foods consumed by the child in the 24 hours before the survey. Colour figure can be viewed at http://wileyonlinelibrary.com

## Notes

1 See Stevens *et al*. (2013) for worldwide anaemia rates.

2 Similarly, in a randomised control trial in India, Bobonis *et al*. (2006) found that a combination of nutrient supplementation (iron and vitamin A) and deworming medications had a positive – though statistically insignificant – effect on haemoglobin levels. Bobonis *et al*. (2006) was not designed to separately identify the effects of iron supplementation and intestinal parasite (deworming) medicine.

3 Hookworms are spread by skin contact with human faecal matter. The eradication efforts featured a deworming campaign and, later, technical assistance in constructing sanitary latrines to arrest the cycle of reinfection.

4 Although India would be a natural setting in which to examine the impact of sanitation on anaemia, the Indian DHS has blood haemoglobin information only in 2005 and does not include district identifiers in that year. Other Indian data sets that would allow us to construct a geographic panel, including the India Human Development Survey and the Indian District Level Health Survey, did not collect haemoglobin information. Therefore, we cannot exploit sanitation policy variation in India for identification as in Spears (2012) or Spears and Lamba (2016) or other natural experiments in the Indian context, as in Geruso and Spears (2017).

5 Miguel and Kremer (2004) is a related study that analyses the effects of school‐level deworming on child school attendance. The authors show that, due to positive treatment externalities, the impact of deworming medicine on school attendance is higher when entire schools, rather than individual children, are dewormed. Our article differs in that we study open defaecation, which is likely to affect haemoglobin through a variety of channels, including – but not limited to – worm infections, and because we study haemoglobin rather than schooling outcomes.

6 Our study also connects to a wide literature on the role of water, sanitation, and disease environment in driving health and human capital accumulation in the developing world and the historical US. See, for example, (Cutler and Miller, 2005; Watson, 2006; Spears, 2012).

7 A strict vegetarian diet, containing no animal proteins, would be lacking in vitamin B12.

8 It is also possible that intestinal diseases spread by open defaecation could affect pregnant women and lead them to pass low iron stores on to their babies. See Allen (1997).

9 Intestinal parasites (helminths) are also commonly called parasitic worms or intestinal worms. Deworming medicine means medicines like albendazole.

10 Children infected with parasites pass them through their bowel movements to the soil; many parasite eggs can live in the soil until they come into contact with new hosts, such as other children who come to the same places to defaecate in the open.

11 It has been documented that enteropathy affects the absorption of vitamin B12 and folic acid, two essential nutrients for the production of haemoglobin (Nath, 2005).

12 See Measure DHS (2013) for more information about collection of haemoglobin data in the field. Kapoor *et al*. (2002) compares the HemoCue${}^{®}$ method to other methods of testing haemoglobin in India. This article uses the haemoglobin measurement that is unadjusted for altitude.

13 The details of the GDP and malaria data construction are described in Appendix A.1.

14 For reference, Figure A1 plots the unconditional relationship between each of these variables and haemoglobin levels. The Figure also shows the unconditional correlation between country malaria rates and haemoglobin levels.

15 In both the 2005/2006 and the 2010/2011 rounds, surveys were conducted between October and April.

16 This WHO standard applies to children 6–60 months of age.

17 There are 25, rather than 26, regions because the western mountain region contains no urban areas. The designation of localities as urban and rural was unchanged between survey rounds.

18 Note that the sample in each year consists of children under 5, so the data regarding literacy and education applies only to relatively recent mothers who will be younger on average. This feature of the data, combined with rapid improvements to education in Nepal over the relevant time period, result in significant differences in reported maternal education and literacy rates between the two survey rounds.

19 Funding nonetheless originated at the national level. See Tayler *et al*. (2005) for more details.

20 Because of the decentralised approach, while the overall goal of reducing open defaecation was a national priority, initiatives undertaken by these local authorities varied significantly in an attempt to tailor approaches to local needs and local behavioural norms. For example, in some places local campaigns consisted of encouraging children to use whistles to call out people as they practised open defaecation. In other localities, access to certain government services was restricted until household members produced verification of latrine/toilet ownership. Additional anecdotal evidence is reported in Knight (2014).

21 Finkelstein (2007) evaluates the impact of the introduction of Medicare in the US by exploiting heterogeneity across regions in the population share of the elderly.

22 If the negative relationship in Figure 3 were driven simply by measurement error, such error should not be correlated with the regional change in our outcome of interest, haemoglobin levels.

23 In Appendix A.2 we present additional results from a complementary IV strategy that exploits the same source of variation as the difference‐in‐differences results in Table 2 but does so by instrumenting for PSU‐level sanitation (${\bar{OD}}_{prt}^{PSU}$) with region × survey round variation.

24 It is yet unknown whether contact with one's own faeces is an important driver of sanitation‐related health outcomes relative to the size of the public good impacts of sanitation, though some helminths including hookworm have a lifecycle that can exploit self re‐infection. Nor is it known whether own OD significantly interacts with neighbourhood OD to affect health, though Geruso and Spears (2017) provide some suggestive evidence against the interaction hypothesis. It is plausible that the specifications in Table 2 that control for own OD (columns (2), (3), (5), and (6)) may over‐control, leading to underestimates of the effects of improved sanitation. Indeed, these specifications do reveal smaller coefficients than in columns (1) and (4) of Table 2, which do not control for own OD, or compared to the results in Table 3 that aggregate to regional means and therefore do not control for own OD.

25 These dwelling controls include indicators for type of wall materials (9 categories), roof materials (8 categories), and floor materials (6 categories) that make up the child's dwelling.

26 Improved water sources include piped water, protected wells, and protected springs.

27 See Spears *et al*. (2013) for an illustration of the statistical power loss resulting when height is dichotomised at a ‘stunting’ threshold in lieu of using the continuous measure.

28 The first stage is reported in Table A3.

29 Intuitively, these 25‐observation regressions coarsen the data to the maximal possible extent.

30 We examine maternal iron supplementation during pregnancy, for which was data collected in both the 2006 and 2011 rounds of the Nepal DHS. Child iron supplementation is far less common. Data on child iron supplementation were only collected in the 2011 round, in which the mean usage rate was 0.028 – i.e. 2.8% of sampled children.

31 The epidemiological literature is not clear on whether mother's in utero iron supplementation continues to have an effect on children's haemoglobin levels by six months of age. Nonetheless, the test is still informative because it can reveal whether our identifying variation is correlated with changes in behaviour or beliefs regarding iron supplementation. Further, under the assumption that babies whose mothers took supplements are likely to be born healthier and, therefore, less susceptible to disease that could block nutrient absorption or deplete stores, there could be an indirect effect on children's haemoglobin.

32 It is also possible that parents could reinforce the impacts of OD changes. In that case, estimates would overstate the effects of sanitation improvements, holding all else constant.

33 The identification strategy of this article is not designed to estimate the causal effect of the diet and medicine on haemoglobin. Therefore, the coefficients on the food and medicine variables reported in the Table A6 regressions should be interpreted with caution. For example, iron supplementation might be an endogenous response to a pregnant mother learning that she is anaemic.

34 Such a possibility is somewhat mitigated by the fact that the classes of factors influencing anaemia in the prior economic, medical, and epidemiological literature is a small set and we have provided evidence on each class of causes in Table 4. However, there could be an omitted variable unknown in the prior literature.

35 These variables are included in the time‐varying region controls.

36 The definition of improved water sources follows the definition in the UNICEF/WHO Joint Monitoring Programme for Water Supply and Sanitation and includes piped water, protected wells and protected springs.

37 The coefficient estimates implied by Figure 1 range from about 0.10 g/dL per 10 percentage point reduction in open defaecation (panel (*a*)) to about 0.20 g/dL (panel (*b*)). In comparison, the within‐region econometric results reported in column (9) of Table 2 imply an effect of 0.196 g/dL per 10 percentage point reduction in open defaection.

38 The SHINE trial, currently underway in Zimbabwe, aims to demonstrate the link between the sanitation environment, environmental enteropathy and anaemia. See Humphrey (2014).

## Appendix A Additional Results

### A.1. Data Used in Section 1

For the motivating analysis in Section 1, data come from several sources. Anaemia and open defaecation data used to construct Figure 1 come from the Demographic and Health Surveys of 45 countries and 18 survey years, spanning 1995–2012. A complete listing of the country‐years is provided in Table A1. Each of the country‐years was matched with time invariant data from the World Bank on land area (World Bank, 2013) and with time variant data from the Penn World Tables on population (Heston *et al*., 2012). These data were used to construct an estimate of the number of people who defaecate in the open per Square Kilometre. The estimate of open defaecation per Square Kilometre is derived by multiplying the fraction of households that defaecate in the open by the population of the country in the year of interest, dividing by the land area in kilometres and taking the log.^39^ The control for GDP *per capita* is time variant and is also taken from the Penn World Tables. GDP *per capita* is in US dollars, converted using the Laspeyere's PPP conversion (Heston *et al*., 2012).

**Table A1:** Demographic and Health Surveys Used in Figure 1

Sub‐Saharan Africa		East Asia	
Angola	2006	Cambodia	2000
Angola	2011	Cambodia	2005
Benin	2001	Cambodia	2010
Benin	2006	East Timor	2009
Burkina Faso	2003		
Burkina Faso	2010	Europe	
Burundi	2010	Albania	2008
Cameroon	2004	Armenia	2000
Cameroon	2011	Armenia	2005
Congo (Brazzaville)	2005	Moldova	2005
DRC	2007	Ukraine	2011
Ethiopia	1997		
Ethiopia	2003	Latin America	
Ghana	2003	Bolivia	1998
Ghana	2008	Bolivia	2003
Guinea	2005	Bolivia	2008
Lesotho	2004	Guyana	2009
Lesotho	2009	Haiti	2000
Liberia	2008	Haiti	2005
Liberia	2011	Honduras	2005
Madagascar	2003	Peru	1996
Madagascar	2008	Peru	2000
Madagascar	2011	Peru	2003
Malawi	2004		
Malawi	2010	North Africa	
Malawi	2012	Egypt	2005
Mali	2001		
Mali	2006	South Asia	
Mozambique	2011	Bangladesh	2011
Niger	2006	India	1998
Rwanda	2005	India	2005
Rwanda	2007	Nepal	2006
Rwanda	2010	Nepal	2011
Sao Tome and Principe	2008		
Senegal	2005	West Asia	
Senegal	2008	Azerbaijan	2006
Senegal	2010	Jordan	2002
Sierra Leone	2008	Jordan	2007
Swaziland	2006	Jordan	2009
Tanzania	2004	Kazakhstan	1995
Tanzania	2007	Kazakhstan	1999
Tanzania	2009	Kyrgyz Republic	1997
Tanzania	2011	Uzbekistan	1996
Uganda	2000		
Uganda	2006		
Uganda	2009		
Zimbabwe	2005		
Zimbabwe	2010		

*Notes*. The Table lists the country‐year observations used in Figure 1. Data on childrens’ haemoglobin and household open defaecation within these 81 Demographic and Health Surveys were collapsed to country‐year means. The surveys included cover 45 countries and span 18 years, 1995–2012.

The control for malaria exposure is a time invariant estimate of malaria incidence in children under five generated by Korenromp (2005) for the WHO. For Africa, these estimates were generated based on 22 longitudinal studies from populations with no access to malaria prevention. The studies defined malaria as fever with malaria parasitemia on a blood slide. In some countries in southern Africa, national malaria case notification rates were used. Korenromp (2005) points out that for countries outside of Africa, there were fewer longitudinal studies on which to base the malaria estimates.

### A.2. Complementary IV Approach to D‐in‐D Results of Table 2

Including neighbourhood fixed effects in Table 2 is not possible because the DHS sampling scheme did not re‐interview in the same neighbourhoods in the 2006 and 2011 rounds. Therefore, in order to narrow the geographic unit of the sanitation environment to the neighbourhood while still exploiting only the within‐region, over‐time variation on display in Figure 2, we additionally present results from a complementary IV strategy. We instrument for PSU‐level sanitation (${\bar{OD}}_{prt}^{PSU}$) with region × survey round variation:

(A.1) $$Hb_{iprt}=\beta_{1}{\hat{\bar{OD}}}_{prt}^{PSU}+\alpha_{2011}+\sum_{r\in R}\alpha_{r}r+f\left(X_{iprt}\right)+\epsilon_{iprt};$$

(A.2) $${\hat{\bar{OD}}}_{prt}^{PSU}=\lambda_{1}{\bar{OD}}_{rt}+\gamma_{2011}+\sum_{r\in R}\gamma_{r}r+g\left(X_{iprt}\right)+\mu_{iprt}.$$

Here, the redundant superscript *PSU* is included to emphasise that open defaecation means are taken at the PSU level (co‐located clusters of approximately 100–200 households). Projecting PSU‐level sanitation, ${\bar{OD}}_{prt}^{PSU}$, onto region‐level sanitation, ${\bar{OD}}_{rt}$, avoids identifying estimates partially off of cross‐sectional differences across PSUs within a region × year. Intuitively, while the IV projection in (A.2) controls for any PSU‐level variation within a region‐year, it still allows the sanitation variable (*OD*) to be defined at the theoretically relevant level of the neighbourhood. This instrumentation strategy also addresses potential measurement error problems that could otherwise attenuate our estimates. Note that (A.1) and (A.2) do not include controls for own open defaecation. Own OD can be a significant share of ${\bar{OD}}_{prt}^{PSU}$ for smaller PSUs, so that a change in ${\bar{OD}}_{rt}$ generates a less than a one‐for‐one change in ${\bar{OD}}_{prt}^{PSU}$, mechanically shrinking the first stage and therefore inflating the IV estimates (Figure A1, Tables A1, A2, A3, A4, A5, A6, A7, A8).

**Table A2:** Summary Statistics for Subsample of Last‐born Children

	Iron sample			
	2006 Round		2011 Round	
	Mean	SD	Mean	SD
	(1)	(2)	(3)	(4)
Child haemoglobin (g/dL)	11.00	1.45	11.11	1.40
Child anaemia, mild (<11 g/dL)	0.47	0.50	0.43	0.50
Child anaemia, moderate (<10 g/dL)	0.23	0.42	0.19	0.39
child anaemia, severe (<7 g/dL)	0.01	0.08	0.00	0.07
Child age in months	28.59	14.77	29.68	15.13
Child male	0.53	0.50	0.53	0.50
Household open defaecation	0.57	0.50	0.43	0.49
Household sanitation (1 ‐ open defaecation)	0.43	0.50	0.57	0.49
Household electricity	0.42	0.49	0.70	0.46
Household improved water source	0.79	0.41	0.87	0.33
Household has health card	0.27	0.44	0.30	0.46
Household urban	0.22	0.42	0.21	0.41
Mother some education	0.40	0.49	0.55	0.50
Mother literate	0.49	0.50	0.62	0.48
Child took parasite medication, last 6 months	0.71	0.45	0.75	0.43
Mother took iron supplements during pregnancy	0.53	0.50	0.78	0.42
Observations	3,191		1,529	

*Notes*. The Table displays summary statistics for the 2006 and 2011 rounds of the Nepal Demographic and Health Survey (DHS), calculated over the subsample of children who were the last children in the data born to their mothers (i.e. no older siblings). This is the sample for which information was collected on the mother's use of iron supplementation during pregnancy. See Table 1 for additional notes.

**Table A3:** Regressions Corresponding to Figure 3 and to the Table 3 First Stage

Dependent variable:	Change in Mean OD within Region, 2006 to 2011			
Sample:	Figure 3 Regressions		IV First Stage (Table 3)	
	Rural Only	Urban Only	Full	Full
	(1)	(2)	(3)	(4)
Exposure (Region mean OD in 2006)	−0.45[Link]	−0.45[Link]	−0.27[Link]	−0.46[Link]
	(0.13)	(0.10)	(0.07)	(0.09)
Urban				X
Change in mean electrification within region				X
Change in mean improved water source within region				X
F‐statistics	12.4	19.9	16.3	25.3
Observations	13	12	25	25

*Notes*. The Table reports results from a series of OLS regressions in which the dependent variable is the change in mean open defaecation within the region from 2006 to 2011. Columns (1) and (2) correspond to Figure 3. Columns (3) and (4) comprise the first stage of the IV analysis in Table 3. Observations are regions. Robust standard errors are displayed. +p < 0.1, *p < 0.05, **p < 0.01.

**Table A4:** Alternative Standard Errors for Coefficients in Columns (4)–(6) of Table 2

Dependent variable:	Haemoglobin (g/dL)		
Specification:	D‐in‐D		
	T2 ‐ Col 4	T2 ‐ Col 5	T2 ‐ Col 6
	(1)	(2)	(3)
Region mean OD	−1.53**	−1.19*	−1.27*
	(0.49)	(0.48)	(0.50)
Survey round FEs	X	X	X
Age‐in‐month × sex indicators	X	X	X
Own OD		X	X
Economic controls			X
Mother education controls			X
Parasite medicine			X
Time‐varying region controls			X
Region FEs	X	X	X
Mean of dependent variable	11.2	11.2	11.2
Observations	6,464	6,464	6,464
Alternative p‐values			
1. Standard clustering by PSU (538 clusters)	<0.01	0.02	0.01
2. Standard clustering by region × year (50 clusters)	<0.01	<0.01	<0.01
3. Wild Cluster Bootstrap by region × year (50 clusters)	0.01	0.03	0.08
4. Standard clustering by region (25 clusters)	<0.01	<0.01	<0.01
5. Wild Cluster Bootstrap by region (25 clusters)	0.01	0.05	0.13

*Notes*. The Table reports results from a series of OLS regressions. The dependent variable in all columns is the child's haemoglobin level. Controls are as described in the Table 2 notes. Observations are children. Standard errors shown in parentheses are clustered at the PSU level. Here p‐values associated with clustering at alternative levels of aggregation are displayed in the last five rows of the Table. We follow the wild cluster bootstrap methodology of Cameron and Miller (2015) in rows labelled (3) and (5), which incorporates an asymptotic refinement. Each of the Wild Cluster Bootstrap calculations in the Table is generated by 5,000 repetitions.

**Table A5:** Main Results Repeated with Binary Anaemia Measures

Dependent variable:	Anaemia, mild or worse (<11 g/dL)		Anaemia, moderate or worse (<10 g/dL)		Anaemia, severe (<7 g/dL)	
Specification:	Cross‐section	D‐in‐D	Cross‐section	D‐in‐D	Cross‐section	D‐in‐D
Sample:	Full	Full	Full	Full	Full	Full
	(1)	(2)	(3)	(4)	(5)	(6)
Region mean OD	0.189[Link]	0.220	0.182[Link]	0.382[Link]	0.011+	0.021
	(0.072)	(0.161)	(0.053)	(0.108)	(0.007)	(0.017)
Survey round FEs	X	X	X	X	X	X
Age‐in‐month × sex indicators	X	X	X	X	X	X
Own OD	X	X	X	X	X	X
Economic controls	X	X	X	X	X	X
Mother education controls	X	X	X	X	X	X
Food and medicine controls	X	X	X	X	X	X
Time‐varying region controls	X	X	X	X	X	X
Region FEs		X		X		X
Mean of dependent variable	0.42	0.42	0.19	0.19	0.01	0.01
Observations	6,464	6,464	6,464	6,464	6,464	6,464

*Notes*. The Table reports results from OLS regressions that repeat specifications from Table 2 with alternative dependent variables. Here, the dependent variables are indicators for anaemia (blood haemoglobin below a threshold level) in place of the continuous haemoglobin measure. The three binary measures indicated at the column headers correspond to the World Health Organization standard thresholds for mild (<11 g/dL), moderate (<10 g/dL), and severe (<7 g/dL) anaemia. The specifications in columns (1), (3), and (5) match the specification in column (3) of Table 2. The specifications in columns (2), (4), and (6) match the specification in column (6) of Table 2. See the Table 2 notes for additional information. Observations are children. Standard errors are clustered at the PSU level. +p < 0.1, *p < 0.05, **p < 0.01.

**Table A6:** Main Results Replicated in the Subsample of Children for Whom Nutrition Data Were Collected

Dependent variable:	Haemoglobin (g/dL)							
Specification:	Pooled cross‐section		D‐in‐D		Pooled cross‐section		D‐in‐D	
Sample:	Food	Food	Food	Food	Iron	Iron	Iron	Iron
	(1)	(2)	(3)	(4)	(5)	(6)	(7)	(8)
Region mean OD	−1.01**	−0.92**	−1.28*	−1.08+	−1.02**	−1.03**	−2.06**	−1.75**
	(0.18)	(0.26)	(0.58)	(0.57)	(0.17)	(0.25)	(0.47)	(0.48)
Parasite medication	0.11+	0.00	0.05	0.03	0.10+	−0.01	0.03	0.01
	(0.06)	(0.06)	(0.06)	(0.06)	(0.05)	(0.05)	(0.05)	(0.05)
Mother iron supplements during pregnancy					−0.04	−0.02	0.08+	−0.02
					(0.05)	(0.05)	(0.04)	(0.05)
Dietary diversity	0.06+	0.03	0.04	0.02				
	(0.03)	(0.03)	(0.03)	(0.03)				
Fruit and vegetables, last 24 hours	−0.02	−0.01	0.00	0.01				
	(0.07)	(0.06)	(0.06)	(0.06)				
Meat and eggs, last 24 hours	−0.08	−0.01	−0.02	0.00				
	(0.07)	(0.06)	(0.06)	(0.06)				
Meal count, last 24 hours								
1 meal	0.16	0.01	0.06	0.00				
	(0.24)	(0.23)	(0.22)	(0.21)				
2 meals	−0.01	−0.16	−0.16	−0.16				
	(0.14)	(0.14)	(0.13)	(0.13)				
3 meals	0.01	−0.13	−0.15	−0.16				
	(0.13)	(0.14)	(0.13)	(0.13)				
4 meals	0.12	−0.05	−0.02	−0.06				
	(0.14)	(0.14)	(0.13)	(0.14)				
5 meals	0.00	−0.12	−0.09	−0.13				
	(0.15)	(0.16)	(0.15)	(0.15)				
6 meals	0.10	−0.04	0.04	−0.04				
	(0.17)	(0.17)	(0.16)	(0.16)				
7 meals	0.07	0.00	0.02	−0.06				
	0.190	0.190	0.180	0.190				
Survey round FEs	X	X	X	X	X	X	X	X
Age‐in‐month × sex indicators	X	X	X	X	X	X	X	X
Own OD		X		X		X		X
Economic controls		X		X		X		X
Mother education controls		X		X		X		X
Time‐varying region controls		X		X		X		X
Region FEs		X	X	X		X	X	X
Mean of dependent variable	11.0	11.0	11.0	11.0	11.0	11.0	11.0	11.0
Observations	4,348	4,348	4,348	4,348	4,720	4,720	4,720	4,720

*Notes*. The Table reports results from a series of OLS regressions. The results are robust to the inclusion of controls for food intake and a control for mother's use of iron supplements during the child's in utero period. The dependent variable in all columns is the child's haemoglobin level. Food controls include an indicator for the consumption of fruits and vegetables within the last 24 hours, an indicator for the consumption of meat and eggs within the last 24 hours, the count of different kinds of foods consumed in the last 24 hours (dietary diversity), and the number of meals (8 categories as indicators, zero meals as the omitted category) within the last 24 hours. Other controls are as described in the Table 2 notes. Observations are children. Standard errors are clustered at the PSU level. +p < 0.1, *p < 0.05, **p < 0.01.

**Table A7:** Parallel Trends: Other Changes in Development Indicators are Uncorrelated with Sanitation Improvement Across‐time, Within‐region

Dependent variable:	Change in region mean of:		
Specification:	Household electrification	Access to improved water source	Family has health card
	OLS	OLS	OLS
	(1)	(2)	(3)
Change in mean OD within region	0.16	−0.21	−0.15
	(0.30)	(0.25)	(0.10)
Observations (regions)	25	25	25

*Notes*. The Table reports results from a series of OLS regressions. The dependent variables are listed in the column headers and are changes over time in regional means of: household electrification; household access to ‘improved’ water sources, where ‘improved’ is defined by the World Bank and UNICEF's Joint Monitoring Programme. Improved water sources include piped water, protected wells and protected springs; and household possession of a national health card. The sole regressor in each column is the change in regional mean open defaecation ($\Delta {\bar{OD}}_{rt}$). Observations are the 25 regions, defined by 13 geographic areas interacted with an urban indicator. Robust standard errors are displayed. +p < 0.1, *p < 0.05, **p < 0.01.

**Table A8:** IV Results

Dependent variable:	Haemoglobin (g/dL)	
Specification:	IV	
Sample:	Full	Full
	(1)	(2)
Panel (*a*): IV regression		
PSU mean OD	−1.51**	−1.63**
	(0.52)	(0.61)
Panel (*b*): correponding first stage regression		
Region mean OD	1.01**	0.90**
	(0.25)	(0.21)
Survey round FEs	X	X
Age‐in‐month × sex indicators	X	X
Own OD		
Economic controls		X
Mother education controls		X
Parasite medicine		X
Time‐varying region controls		X
Region FEs	X	X
Mean of dependent variable	11.2	11.2
First stage F‐statistics	16.7	19.2
Observations	6,464	6,464

*Notes*. The Table reports results from IV regressions that instrument for PSU‐level mean open defaecation (${\bar{OD}}_{prt}^{PSU}$) with regional mean open defaecation (${\bar{OD}}_{rt}$). Panel (*a*) reports the IV results, and panel (*b*) reports the corresponding first stages. Controls are as described in the Table 2 notes. Observations are children. Standard errors are clustered at the PSU level. +p < 0.1, *p < 0.05, **p < 0.01.

Table A8 reports results from the IV approach. Panel (*a*) reports effects instrumenting for PSU‐level open defaecation with ${\bar{OD}}_{rt}$. First stage results for the IV analysis are reported in panel (*b*). The IV results are statistically indistinct from the corresponding estimates in Table 2.

### A.3. Inference Under Alternative Clustering

For the main results in Table 2, we cluster standard errors at the level of the PSU. The difference‐in‐differences analysis in columns (4)–(6) uses variation in mean open defaecation at the level of the region‐by‐year. Therefore, as an alternative to the PSU‐level clustering, we present inference using clustering at the geographically more aggregated levels of the region‐by‐year and region.

Table A4 lists p‐values for our coefficient estimates for each of the following clustering alternatives: by PSU (538 clusters) by region × year (50 clusters) and by region (25 clusters). As Cameron and Miller (2015) discuss, standard clustering techniques tend to over‐reject the null for small numbers of clusters. ‘Small’ is generally considered to be less than 50 (Bertrand *et al*., 2004; Cameron *et al*., 2008). Therefore, we follow the wild cluster bootstrap methodology of Cameron and Miller (2015), which incorporates an asymptotic refinement to address this issue.^40^ The bottom five rows of Table A4 report a complete set of p‐values based on the three alternative levels of clustering and include results generated by both standard and wild cluster bootstrap methods. In 13 of 15 cases, the p‐values are ≤ 0.05 and in many cases they are ≤ 0.01.

Finally, Table 3 complements this clustering analysis by taking a completely separate approach to statistical inference. We collapse the data to 25 observations and regress within‐region mean differences in haemoglobin on within‐region mean differences in open defaecation over the 2006–11 study period. These regression results, which are statistically significant at p < 0.01, are not subject to the clustering issue discussed above. Robust standard errors in Table 3 are consistent without asymptotic refinement. (Intuitively, these 25‐observation regressions coarsen the data to the maximal possible extent.)
